# Room Temperature Phosphorescent (RTP) Thermoplastic Elastomers with Dual and Variable RTP Emission, Photo‐Patterning Memory Effect, and Dynamic Deformation RTP Response

**DOI:** 10.1002/advs.202103402

**Published:** 2021-12-23

**Authors:** Yuefa Zhang, Qikun Sun, Lingtai Yue, Yaguang Wang, Shuaiwei Cui, Haichang Zhang, Shanfeng Xue, Wenjun Yang

**Affiliations:** ^1^ Key Laboratory of Rubber‐Plastics of Ministry of Education/Shandong Provincial Key Laboratory of Rubber‐Plastics School of Polymer Science & Engineering Qingdao University of Science &Technology Qingdao China

**Keywords:** dual room temperature phosphorescence, phosphorescent polymers, photo‐patterning memory effect, thermoplastic elastomers, versatile strategy for RTP polymer

## Abstract

Room temperature phosphorescent (RTP) polymers have advantages of strength, toughness, and processing and application flexibility over organic small molecular crystals, but the current RTP polymers are all from rigid plastics and involve chemical linkage and hydrogen and ionic bonds, and thermoplastic RTP elastomer has not been attempted and realized. Moreover, solution‐processed films by simply mixing polymers and organic RTP materials can only show weak and single blue RTP. Here it is presented that such elastomer films, once thermomechanically plasticized, can emit bright and long‐lived dual RTP. Moreover, they exhibit photo‐activation memory effect, variable RTP colors and dynamic deformation RTP response. These results reveal that thermoplasticizing has altered the dispersion states and micro‐environment of RTP molecules in matrix, and the cohesion of elastic polymer itself can also greatly restrict non‐radiative relaxations to boost both blue mono‐molecular and yellow micro‐crystalline RTP. This work provides an effective and versatile processing strategy for tuning and enhancing the RTP properties of doped RTP polymers.

## Graphical abstract

Organic room temperature phosphorescent (RTP) molecules are doped into thermoplastic elastomer by thermoplasticizing solution‐processed films, which alters dispersed state and micro‐environment of RTP molecules and contributes variable dual RTP emission, photo‐pattern memory effect, and deformation RTP response. This is a versatile strategy to enhance and tune photoluminescence (PL) properties of RTP polymers.

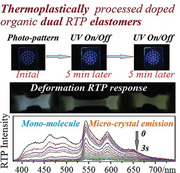

## 1 Introduction

Some inorganic salts such as SrAl_2_O_4_ and CaAl_2_O_4_ doped with Eu, Nd, and Dy ions can exhibit long‐lived room temperature phosphorescence (RTP) and have been used for displaying, information storage, anti‐counterfeiting, glow‐in‐the‐dark products, and bio‐imaging.^[^ ^1^, ^2^, ^3^, ^4^, ^5^ ^]^ However, they require rare earth elements and are usually powdery and toxic. Alternatively, organic RTP materials are being developed in the past years because of advantages of low cost, bio‐compatibility, structure diversity, and modification flexibility and good process‐ability.^[^ ^6^, ^7^, ^8^ ^]^ Up to now, a variety of approaches, such as introduction of hetero atoms and heavy halogens,^[^ ^9^, ^10^ ^]^ crystallization engineering,^[^ ^11^, ^12^ ^]^ doping in the rigid matrix,^[^ ^13^, ^14^, ^15^ ^]^ H‐aggregation,^[^ ^16^, ^17^ ^]^ and so forth, are proposed for obtaining organic RTP materials. However, their mainstay is still organic small molecular crystals that are more fragile and poor strength and hard to fabricate products with any shape and size, hindering many practical applications. As we know, general polymers are characteristic of excellent strength, toughness, and process‐ability, thus, it is very meaningful and necessary to develop long‐lived RTP polymers, and recently, an increasing interest in such RTP polymers is emerging and has advanced relevant development and understanding.^[^ ^18^, ^19^, ^20^, ^21^ ^]^

However, the existing commercial polymers are basically non‐phosphorescent especially at room temperature. To obtain RTP polymers, introduction of triplet chromophores and suppression of non‐radiative relaxation are two key factors in enhancing RTP. Introducing organic heterocycles with RTP potential into polymers by grafting and random co‐polymerization and cross‐linking has been reported more, but there is still a formidable challenging to provide a reasonable strategy to manipulate polymer structure and composition because these chemical modifications are not very effective in achieving ultralong RTP.^[^ ^22^, ^23^ ^]^ There are several successful examples that mainly depend on the inter‐molecular hydrogen and ionic bonding to restrict the relaxation motions of chromophores.^[^ ^24^, ^25^ ^]^ It should be noted that the introduction of bulky heterocycles and/or the presence of abundant hydrogen and ionic bonds can damage the thermoplasticizing, and such polymers can usually be solution‐processed into films at most, losing the advantage that thermoplastic polymers can be processed into products of any shape and size. Moreover, polymers with abundant ionic and hydrogen bonds are readily hygroscopic and show unstable RTP properties. On the other hand, the existing investigations mainly emphasize the importance of polymer rigidity and special interactions between RTP moiety and polymer matrix but focus little attention on the innovation of the processing approaches. PVA (polyvinyl alcohol) and PMMA (poly(methyl methacrylate)) are the most widely used matrixes. It is meaningful and necessary to enable other common thermoplastic polymers and even elastomers to become admirable RTP matrixes without complicated chemical modification because this will greatly expand the scope of RTP materials and help understand RTP mechanisms.

## 2 Results and Discussion

**Figure 1:**
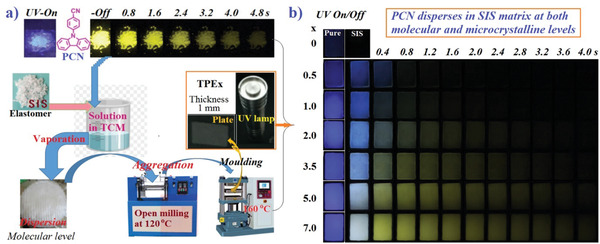
a) The diagram for the preparation procedure of doped RTP elastic polymers (TPEx) consisting of *N*‐(4‐cyanophenyl)carbazole (PCN) and styrene‒isoprene‒styrene block copolymer (SIS), where *x* represents the doping mass (g) in 100 g of SIS). b) PL photographs of TPEx under ambient conditions before and after removing 365 nm light. The excitation duration time is10 s.

Mixing the existing organic RTP crystals (such as commercial carbazole derivatives) into thermoplastic polymers by solution‐processing or thermomechanically blending is the simplest doping way. However, as expected, neither of the two methods is effective to achieve impressive RTP properties. We think that the solvent residue and the plenty of nanopores inevitably exist in the solution‐processed films, which can aggravate the thermal deactivation and oxygen quenching of molecularly dispersed RTP molecules. In the direct thermal blending, RTP crystals will be broken into smaller grains and unevenly dispersed in polymer matrix, which contributes to the approximate RTP properties as the original crystals at best. On the basis of the above views, we consider that the combination of solution processing and thermomechanically plasticizing may be a promising route to long‐lived thermoplastic RTP polymers because this will enhance the role of strong cohesion of polymer and change the dispersion states and micro environment of RTP molecules to reduce thermal deactivation and manipulate RTP properties. To validate the effectiveness, here we use styrene‒isoprene‒styrene block copolymer (SIS) as the matrix and *N*‐(4‐cyanophenyl)carbazole (PCN) as the dopant to develop, for the first time, thermoplastic RTP elastomer (TPEx, *x* represents the doping mass (g) of PCN in 100 g of SIS). PCN can be readily synthesized from commercial carbazole and 4‐fluorobenzonitrile and has been investigated widely.^[^ ^26^, ^27^ ^]^ PCN crystal prepared here emits prompt deep blue fluorescence and delayed yellow RTP with a lifetime of 365.1 ms under 365 nm UV light excitation (Figure S1, Supporting Information). Polar PCN and non‐polar SIS are all soluble in chloroform (TCM), and the solution‐processed films emit weak and short blue RTP afterglow after drying and irradiation with sustaining UV light (Figure S2, Supporting Information). This delayed blue emission is ascribed to the mono‐molecular RTP because its crystal only emits yellow RTP with the main peak at 545 nm. Further, we thermoplasticize the above films at 120 °C on open mill for 2‒3 min and then mold them into 1 mm thick rectangle sheets (TPEx). A general processing procedure is diagrammed in **Figure** **1**a. Unexpectedly, TPEx can emit long RTP afterglow, once continuously photo‐activated for 5‒8 s by UV light (Figure 1b). Moreover, the higher the content of PCN, the shorter the photo‐activation time required, the stronger the intensity of afterglow and the longer the delay time of afterglow. Interestingly, variable RTP colors are observed with both the delay time and the content of PCN, revealing that thermoplasticizing has resulted in the partial re‐crystallization of PCN molecules and enabled the dual RTP emission from both mono‐molecules and micro‐crystals. Notably, this dual RTP emission is easily achieved by combining solution processing and thermal blending but hard to produce via single solution processing or direct solid blending. A sustaining irradiation time is required for the appearance of RTP, which is ascribed to the existence of oxygen in SIS matrix. The long afterglow emission signifies that RTP molecules have been confined seriously in an oppressed space by strong cohesion and dense packing of the compacted polymers, which can greatly restrict the non‐radiative relaxations to boost RTP since there are not strong and special interactions between PCN and SIS molecules in principle.

**Figure 2:**
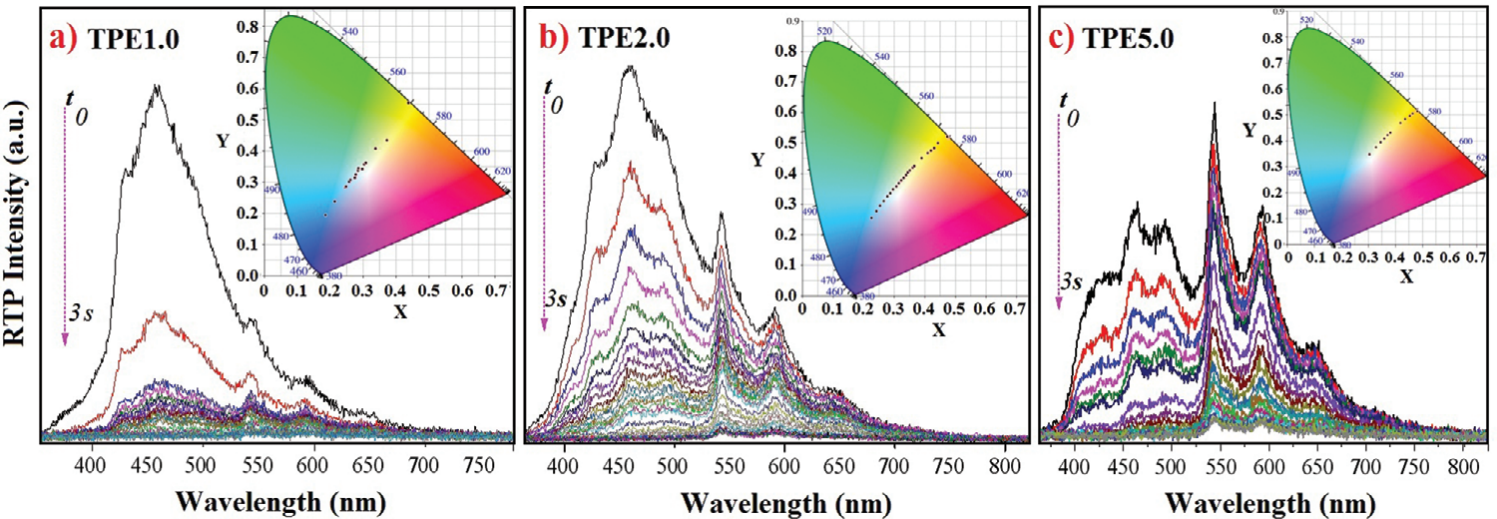
The delayed RTP spectra and the corresponding CIE 1931 chromaticity coordinates of photo‐activated TPEx after 365 nm light excitation. A clear boundary at ≈525 nm is observed, and the emissions before and after 525 nm are from mono‐molecules and micro‐crystals of PCN in SIS matrix, respectively.

All the samples are photo‐activated for 10 s before various measurements, and the generated RTP afterglow can last for 6‒10 s viewed by the naked eyes in the dark with the increase of PCN content. Moreover, TPEx exhibit photo‐activation memory effect and the photo‐activated samples instantly generate RTP after transitory UV irradiation within 10‒15 min. The delayed emission spectra are recorded by a charge‐coupled device (CCD) spectrometer in the dark under 365 nm light excitation. As shown in **Figure** **2** and Figure S3 (Supporting Information), RTP spectra cover the entire visible region ranging from near ultraviolet (380 nm) to near infrared light (750 nm). The two distinctly different emission bands are observed: one appears before 525 nm and the other after. Long‐wave emission band shows a main peak at ≈540 nm and two gradually decreased shoulder peaks at 590 and 640 nm, which is a featured RTP emission of commercial carbazole derivative crystals.^[^ ^28^, ^29^ ^]^ The proportion of delayed blue emission is higher than that of yellow emission in the spectra with low PCN content, but it gradually decreases with the increase of PCN content, demonstrating a gradual increase of aggregated crystallization. Interestingly, we can observe very different decay rates between long‐ and short‐wave emission bands with the boundary at ≈525 nm, and the short‐wave band decreases faster with the time delay. As a result, the variable RTP emission colors and spectra are achieved in two ways. When UV light is just turned off, the RTP afterglow gradually changes from blue to white colors with the increase of PCN content (Figure 1b), which can be verified by the calculated CIE 1931 chromaticity coordinates (Figure 2). When the content of PCN is constant, the Commission Internationale De L'E'clairage (CIE) 1931 chromaticity coordinates continuously change with the delay time, and the variable range depends on the specific content of PCN in SIS. For example, TPE1.0 changes from blue (0.18, 0.19) to white (0.30, 0.34) to light yellow (0.44, 0.56), TPE2.0 from pale blue (0.22, 0.25) to white (0.30, 0.35) to yellow (0.47, 0.52), and TPE5.0 goes from white (0.31, 0.35) to yellow (0.48, 0.52) (Figure 2a‒c, Figure S3, Supporting Information). These results manifest the ability of this processing procedure in tuning RTP properties.

**Figure 3:**
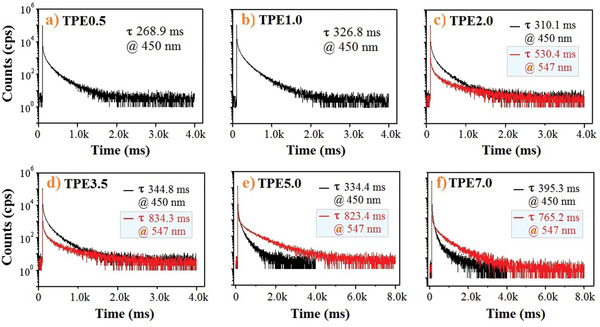
The RTP decay curves and fitted lifetimes of photo‐activated TPEx monitored at 450 and 547 nm and excited at 365 nm.

To assess the dual RTP lifetimes, the time‐resolved PL decay curves are measured for emissions at 450 and 547 nm (**Figure** **3**). The fitted RTP lifetimes for 450 nm blue emission are in the range of 270‒390 ms, which is commendable because it is the mono‐molecular RTP emission in elastomer where there are no special interactions and indicates that the cohesion of elastomer (SIS) has greatly confined non‐radiative relaxations of RTP molecules to boost RTP emission. The fitted RTP lifetimes for TPEx at 547 nm are even longer (530‒830 ms) than that of the original PCN bulk crystal (the decay curves for TPE0.5 and TPE1.0 at 547 nm are not determined due to the weak emission intensity). In principle, the micro‐crystals formed in polymer matrix during thermoplasticizing should be very tiny and imperfect. We have analyzed TPEx by differential scanning calorimeter and X‐ray diffractometer and do not find observable crystal endothermic peaks, and notable crystal diffraction peaks are only observed in TPE7.0 with high PCN content (Figure S4, Supporting Information), evidencing the tininess and imperfection of microcrystals formed during thermoplasticizing. Thermoplasticizing solution‐processed films has created new dispersion states and micro‐environment of RTP molecules to enable unique photo‐physical properties that are hard to achieve for normal crystals, such as long‐lived molecular and microcrystal RTP and dual RTP, etc. Based on the single crystal obtained here, PCN molecules adopt a more twisted (48.1^o^) D‒A motif to pack together and can form multiple strong inter‐molecular CH···N and CH···*π* interactions (Figure S5, Supporting Information). Quantum chemical calculations based on three closely interacted molecules present two possible inter‐system crossing (ISC) channels with an overall probability of 97.4% in terms of the same transition orbital compositions between S_1_ and T_n_ within ±0.3 eV energy level (Figure S5, Supporting Information), and one possible ISC channel with the probability of 10.7% is calculated for mono‐molecule. Nevertheless, the theoretical results support the triplet population of both mono‐molecular and crystalline states.

**Figure 4:**
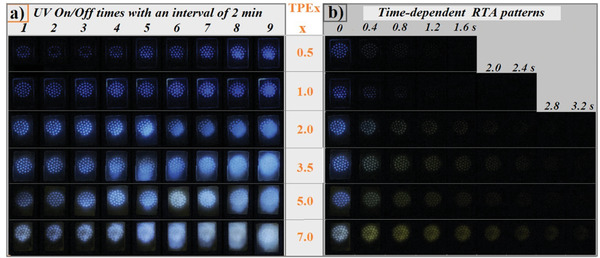
a) The afterglow photographs of photo‐activated (6 s) patterns of TPEx after transitory 365 nm light excitation with an interval of 2 min. b) The delayed afterglow photographs of photo‐activated (6 s) patterns of TPEx after removing 365 nm light.

As mentioned above, the current RTP polymers are based on rigid and polar plastics such as PVA and PMMA. Very recently, Reineke et al. reported a doped PMMA film with ultra‐long RTP by covering an oxygen‐barrier layer (Exceval, ethylene‐vinyl alcohol co‐polymer),^[^ ^30^ ^]^ and Ma et al. developed another doped PMMA film with high RTP efficiency but very short RTP lifetime (10.6 ms).^[^ ^31^ ^]^ These PMMA films produce RTP after a sustaining UV irradiation and are thought to be promising photo‐patterning materials because the all‐or‐nothing luminescent materials when turning on/off UV light are impossible to label and store complex information. The single‐time writing in the amorphous films was realized recently using 254 nm UV light radiation to cross‐link polymers.^[^ ^32^, ^33^ ^]^ However, there are problems such as low contrast image and ultra‐long printing time (>1 h) as well as impossible second image printing after the first one disappeared. SIS is a non‐polar and non‐phosphorescent elastomer but can still serve as a doping matrix to afford admirable thermoplastic RTP polymers through a modified processing procedure. Moreover, the RTP generation of TPEx also requires a certain time of sustaining UV light irradiation and the photo‐activated spots can instantly regenerate RTP after a transient UV light excitation. This provides a time window for both labeling and reading‐out information. Thus, we photo‐activate the samples by covering a lattice mask, and a clear and bright RTP lattice pattern is formed upon 6 s of UV light excitation (**Figure** **4**, Video S1, Supporting Information). We further inspect the memory effect of photo‐activated patterns by flashily turning on/off UV light with an interval of 2 min (Figure 4a). It is observed that TPE0.5 and TPE1.0 are insufficiently and heterogeneously photo‐activated. Interestingly, those dim spots remember that they are ever photo‐activated since the patterns become brighter and clearer as the times of UV on/off increases. TPEx with low PCN content such as TPE0.5 and TPE1.0 can show the clearly discernible patterns within 15 min, and these light spots become larger and joined each other and finally obscure patterns. The time for the photo‐activated pattern to be clearly read becomes shorter with the increase of PCN content, implying that the corresponding samples are more and more easily photo‐activated. Notably, when photo‐activated samples are left at room temperature for 15‒20 min, the photo‐patterns disappear automatically, enabling a repetitive and a new photo‐patterning. Figure 4b displays the changes of photo‐activated patterns with the delayed time. A clear delayed pattern is seen by the naked eyes in the dark, and the lasting time of clear photo‐pattern gradually increases with PCN content. Moreover, the changed colors are observed.

**Figure 5:**
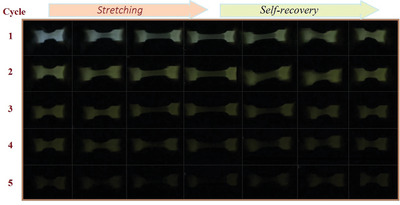
The delayed afterglow photographs of photo‐activated (10 s) dumbbell‐shape specimen of TPE5.0 under successive dynamic deformations (stretching and self‐recovery). The deformed region shows the faster decrease in RTP intensity.

An advantage of thermoplastic polymers is that they can be thermomechanically molded into products of any shape and size. Limited by sample weight, only 1 mm thick rectangle specimens are molded by high‐temperature tablet press to demonstrate the thermoplasticizing process‐ability. Further, the characteristic of an elastomer is that it can be reversibly deformed by mechanical bending and stretching. Thus, a small dumbbell shape specimen is prepared from TPE5.0 and photo‐activated for 10 s, and then dynamically artificial stretching is performed. The recorded RTP afterglow images are depicted in **Figure** **5** and Video S2 (Supporting Information). It is observed that the initial white afterglow gradually becomes weak and yellow with time delay, and the intensity of afterglow in the non‐deformed region is higher than that in the deformed region. Moreover, the large deformation region shows weaker afterglow than the small one in the certain same stretching and self‐recovery cycle. These results indicate that the dynamic deformation can aggravate the vibration inactivation and oxygen quenching. To our knowledge, this is the first example of polymers exhibiting dynamic deformation RTP response, and such materials will find promising applications in the future.

**Figure 6:**
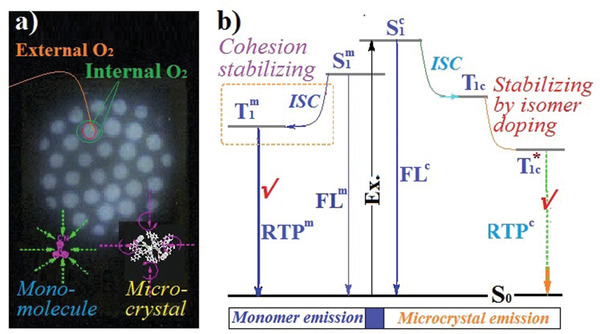
a) The schematic illustration of photo‐activated spot is quenched by external and internal oxygen. It takes time for the re‐entrance and enables photo‐patterning memory effect. Cohesion of polymer plays a more vital role in restricting mono‐molecular motions due to the rigidity of micro‐crystal itself. b) The diagram for the dual RTP emission of PCN doped SIS elastomer.

Based on the above results, we can summarize the dispersion states and PL mechanism of TPEx (**Figure** **6**). PCN molecules dispersed in the solution processed SIS films at molecular level are partially aggregated and re‐crystallized after thermoplasticizing, and the formed micro‐crystals are tiny and imperfect and thus lack the thermal melting and X‐ray diffraction behaviors of crystals but can still exhibit crystal PL properties. Upon photo‐excitation, PCN molecules form mono‐molecular and crystalline singlet states (named S_1_^m^ and S_1_^c^) and undergo ISC to populate the triplet states T_1_^m^ and T_1_^c^. Unlike bulky perfect crystals, the mono‐molecules and micro‐crystals are sensitive to oxygen, and these T_1_ excitons are initially quenched. As the photo‐excitation continues, triplet oxygen in matrix is mostly consumed and T_1_ excitons are accumulated to enable phosphorescence process. The factors affecting photo‐activation time are complex, but the T_1_ population capacity of RTP molecules and the active oxygen density in polymer matrix should play more important roles. The excited states are generally in polar charge transfer states and stabilized by surroundings. This mono‐molecular T_1_^m^ should be stabilized only by cohesion of non‐polar polymer to some extent and emits blue RTP, whereas the micro‐crystalline T_1_^c^ is mainly stabilized by the isomer doping because TPEx emit the featured long‐wave RTP same as PCN crystal. PCN is synthesized from commercial carbazole (CCZ) that contains trace isomer benzo[*f*]indole (Figure S6, Supporting Information), and the RTP generation and feature of CCZ derivative crystals are dominated by the trace isomer doping.^[^ ^34^ ^]^ We have prepared PCN analogue laboratory self‐synthesized *N*‐(4‐cyanophenyl)carbazole (L‐PCN) from the laboratory self‐synthesized carbazole (LCZ) and found that L‐PCN indeed emits very weak crystal RTP (Figure S7, Supporting Information). Therefore, most CCZ‐based RTP crystals and amorphous polymers can be processed into dual RTP‐emitting doped polymers with photo‐patterning memory effect via the innovative processing procedure proposed here. Since the oxygen migration driven by a concentration gradient from the adjacent unactivated regions is slow, the activated regions can instantly emit RTP after being excited again by UV light until the excitons are significantly quenched by the reentered oxygen. After that, a new photo‐patterning can be conducted, enabling repeatable RTP information storage and read out. It can be expected that RTP image can be erased fast and easily if 4 µm of IR light absorbed well by PMMA is applied to heat up samples and facilitate the oxygen refilling.^[^ ^35^, ^36^, ^37^ ^]^ We believe that, with the help of a focused LED spot or a UV laser spot, the fast and precise direct writing without the need of a mask is possible.^[^ ^30^ ^]^

## 3 Conclusion

In summary, the advantages of RTP polymers are the strength, toughness, and process‐ability, but the solution spin‐ and droplet‐coating are still inadequacy in processing and application flexibility and the RTP properties of formed films need to be improved. We have developed a facile and effective processing strategy that can not only improve RTP properties but also tune excitation and emission behaviors. Through thermomechanically plasticized solution‐processed films, the dispersed states and micro‐environment of RTP molecules are tuned and the strong cohesion of polymer can effectively suppress the non‐radiative relaxation to achieve long‐lived RTP amorphous polymers. More importantly, long‐lived thermoplastic RTP elastomers with bright afterglow, tunable dual RTP emission and photo‐patterning memory effect, which are usually not readily available, can also be implemented in the absence of chemical linkage and ionic and hydrogen bonds. This innovative processing strategy is not only crucial on the way to large‐scale applications and easy handling of RTP materials but also expands the scope of long‐lived RTP polymers and inspires a new under‐standing of RTP phenomenon and mechanism.

## Conflict of Interest

The authors declare no conflict of interest.

## Supporting information

Supporting Information

Supporting Information

Supporting Information

## Data Availability

Research data are not shared.
