# Supplementary material for: Room Temperature Phosphorescent (RTP) Thermoplastic Elastomers with Dual and Variable RTP Emission, Photo‐Patterning Memory Effect, and Dynamic Deformation RTP Response
Source: Adv Sci (Weinh). 2021 Dec 23;9(5):2103402. doi: 10.1002/advs.202103402 (PMC8844475; doi:10.1002/advs.202103402)
Supplement: Supplementary file 1 — Supporting Information [file ADVS-9-2103402-s001.pdf]

WILEY-VCH

ADVANCED SCIENCE
Open Access

Supporting Information

for *Adv. Sci.*, DOI: 10.1002/advs.202103402

Room Temperature Phosphorescent (RTP) Thermoplastic Elastomers with Dual and Variable RTP Emission, Photo-patterning Memory Effect, and Dynamic Deformation RTP Response

Yuefa Zhang,[†] Qikun Sun,[†] Lingtai Yue, Yaguang Wang, Shuaiwei Cui, Haichang Zhang, Shanfeng Xue, Wenjun Yang**

Supporting Information

Room Temperature Phosphorescent (RTP) Thermoplastic Elastomers with Dual and Variable RTP Emission, Photo-patterning Memory Effect, and Dynamic Deformation RTP Response

Yuefa Zhang,[†] Qikun Sun,[†] Lingtai Yue, Yaguang Wang, Shuaiwei Cui, Haichang Zhang, Shanfeng Xue, Wenjun Yang**

Key Laboratory of Rubber-plastics of Ministry of Education/Shandong Province (QUST), School of Polymer Science & Engineering, Qingdao University of Science & Technology, 53-Zhengzhou Road, Qingdao, 266042, P. R. China.
E-mail: ywjph2004@qust.edu.cn, sfxue@qust.edu.cn.

Materials

Styrene– isoprene–styrene block copolymer (SIS 1125, 300 mPa·s for 25% toluene solution at 25 °C, the molecular weight is 80,000-300,000) was obtained from Yueyang Baling Huaxing Petrochemical Co., Ltd., 4-Fluorobenzonitrile and 9H-carbazole were obtained from Energy Chemrcal Co., Ltd.

General method

^{1}H and ^{13}C NMR spectra was recorded on a Bruker AC500 spectrometer at 500 MHz and 125 MHz, respectively, using deuterated chloroform or deuterated dimethyl sulfoxide as the solvent and tetramethylsilane (TMS) as the internal standard. High performance liquid chromatography (HPLC) was performed on an Essentia LC-16. The running rate was 1 mL/min. X-ray diffraction (XRD) measurements were determined on a D-MAX 2500(18KW). Differential scanning calorimetry (DSC) curves were determined on a Netzsch DSC (204F1) instrument at a heating rate of 10 °C /min. Photo-fluorescence and phosphorescence emission spectra were recorded on Hitachi F-4600 spectrophotometers. Fluorescence and phosphorescence decay curves were recorded by a Hamamatsu compact fluorescence lifetime spectrometer (FLS-1000). The lifetimes (τ) of the luminescence were obtained by fitting the decay curve with a multi-exponential decay function of

$$R(t) = \sum_{i} B_i e^{-\frac{t}{\tau}} \tag{S1}$$

where B_i and τ_i represent the amplitudes and lifetimes of the individual components for multi-exponential decay profiles, respectively.

The digital photographs were captured by the FDR-AX700 4K HDR digital cameras (SONY, Japan). Photoluminescence spectra and photographs at room temperature were performed on a QE Pro spectrometer with a CCD array (Ocean Optics) as a power detector.

The Gaussian 09 program was utilized to perform the TD-DFT calculations. The ground state (S_0) geometry was obtained from the single crystal structure and no further geometry optimization was conducted in order to maintain the specific molecular configuration and corresponding intermolecular locations. The exciton energies of the n-th singlet (S_n) and n-th triplet states (T_n) were obtained on the corresponding ground state structure using TD-B3LYP/6-31G*. The Kohn–Sham frontier orbital analysis and spin density distributions were obtained in order to elucidate the mechanisms of possible singlet–triplet intersystem crossings (ISC). The possible S_1 to T_n ISC channels are believed to share part of the same transition orbital compositions, and the energy levels of possible T_n are considered to lie within the range of $E_{S1} \pm 0.3$ eV. Especially, the major ISC channels are mainly determined based on two elements. First, the ratio of the same transition configuration in S_1 and T_n should be large in all the transition orbital compositions. Secondly, the energy gap between S_1 and the specific T_n state should be small.

Synthesis and Characterization

The synthetic route of laboratory-self-synthesized carbazole (LCZ).

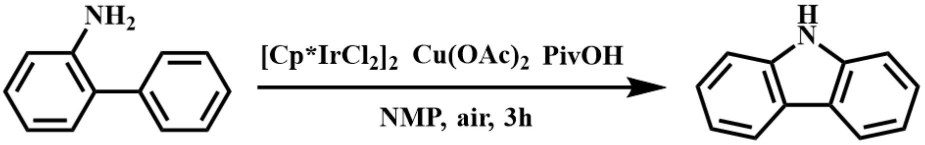

Scheme S1. Synthetic route of LCZ.

9H-carbazole (LCZ). In a 100 mL two-necked flask 2-Aminobiphenyl (2.50 g, 14.77 mmol), $[Cp^*IrCl_2]_2$ (0.25 g, 0.31 mmol), $Cu(OAc)_2$ (0.54 g, 2.97 mmol), and PivOH (3.05 g, 29.86 mmol) in NMP (50 mL) was stirred under air at 120 °C for 3 h. After cooling, the reaction mixture was extracted with ethyl acetate, the combined organic layer dried with anhydrous $MgSO_4$, filtered and concentrated in vacuo. The crude product was purified by silica-gel column chromatography using petroleum ether/dichloromethane (5:1, v/v), yielding a white solid (1.31 g, Yield 53 %). ^{1}H NMR (500 MHz, DMSO-d_6) δ 11.27 (s, 1H), 8.13 (d, *J*

= 7.7 Hz, 2H), 7.51 (d, J = 8.1 Hz, 2H), 7.41 (t, J = 7.6 Hz, 2H), 7.18 (t, J = 7.4 Hz, 2H). ^{13}C NMR (126 MHz, DMSO-d_6) δ 139.66, 125.44, 122.34, 120.08, 118.42, 110.87.

The synthetic route of 4-(9H-carbazol-9-yl)benzonitrile (PCN/L-PCN).

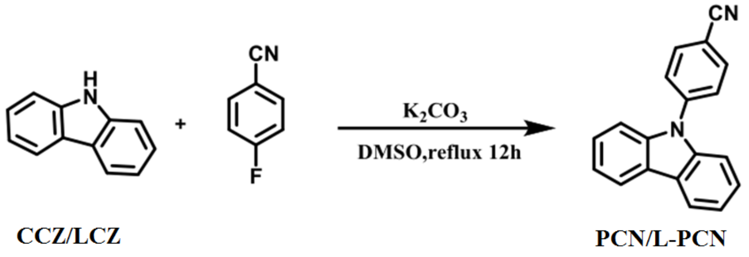

Scheme S2. Synthetic route of PCN.

4-(9H-carbazol-9-yl)benzonitrile (PCN/L-PCN). In a 100 mL bottom, 9H-carbazole (CCZ/LCZ, 3.00 g, 17.94 mmol) and K_2CO_3 (8.22 g, 26.91 mmol) in DMSO (30 ml) was stirred at room temperature for 1 h. 4-Fluorobenzonitrile (2.61 g, 21.55 mmol) was added in the mixture and stirred at 150 ^{o}C for 12 h. The reaction mixture was poured into a large amount of ice water and stirred for additional 1 h. After the reaction mixture was extracted with ethyl acetate, the combined organic layer dried with anhydrous $MgSO_4$, filtered and concentrated in vacuo. The crude product was purified by silica-gel column chromatography using petroleum ether/ethyl acetate (10:1, v/v) as the eluent to give the compound as a white solid (4.09 g, Yield 85 %).

PCN: ^{1}H NMR (500 MHz, Chloroform-*d*): δ 8.14 (d, J = 7.7 Hz, 2H), 7.92–7.86 (m, 2H), 7.75–7.70 (m, 2H), 7.49–7.38 (m, 4H), 7.33 (ddd, J = 8.0, 6.2, 1.9 Hz, 2H). ^{13}C NMR (125MHz, Chloroform-*d*): δ 142.05, 139.87, 133.89, 127.08, 126.35, 123.98, 120.97, 120.55, 118.33, 110.45, 109.49.

L-PCN: ^{1}H NMR (500 MHz, Chloroform-*d*): δ 8.14 (d, J = 7.8 Hz, 2H), 7.94 – 7.88 (m, 2H), 7.78 – 7.71 (m, 2H), 7.48 – 7.41 (m, 4H), 7.34 (ddd, J = 8.0, 6.3, 1.8 Hz, 2H). ^{13}C NMR (126 MHz, Chloroform-*d*): δ 142.08, 139.92, 133.93, 127.11, 126.39, 121.02, 120.60, 118.38, 110.50, 109.54.

Preparation of TPEx(x represents the doping mass (g) of PCN in 100 g of SIS). SIS and PCN were stirred and dissolved in chloroform at room temperature. The resulting solution was evaporated and the formed film was dried in oven at 60 ^{o}C. Then, the films were thermoplasicized on an open two-roll mill at 120 ^{o}C for 2–3 min. Finally, the thermoplasicized film was molded into 1 mm thick sample.

Supplementary Figures and Tables

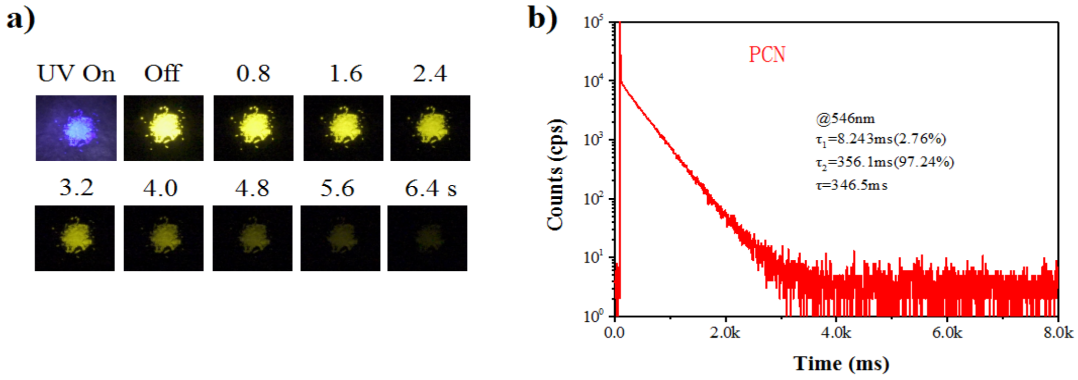

Figure S1. a) PL Photographs of PCN crystal taken before and after removing 365 nm UV light irradiation. b) The RTP decay curve and fitted lifetimes of photo-activated PCN crystal monitored at 546 nm and excited at 365 nm.

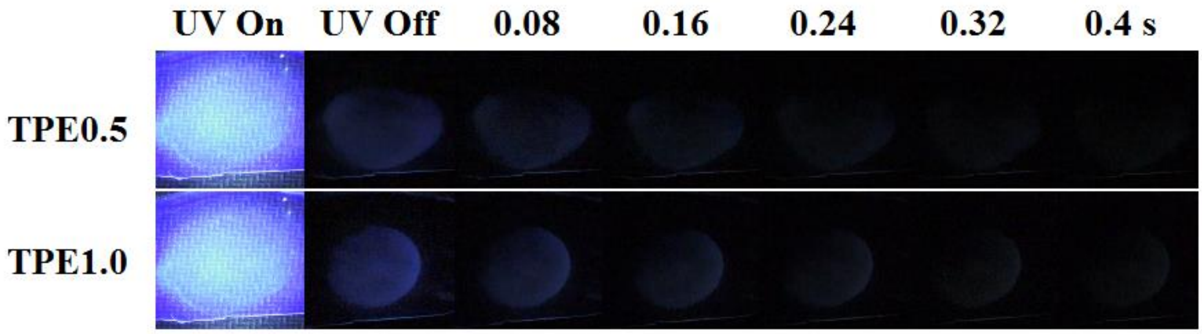

Figure S2. The prompt PL and afterglow photographs of solution-processed films of PCN and SIS in chloroform after photo-activation for 10 s.

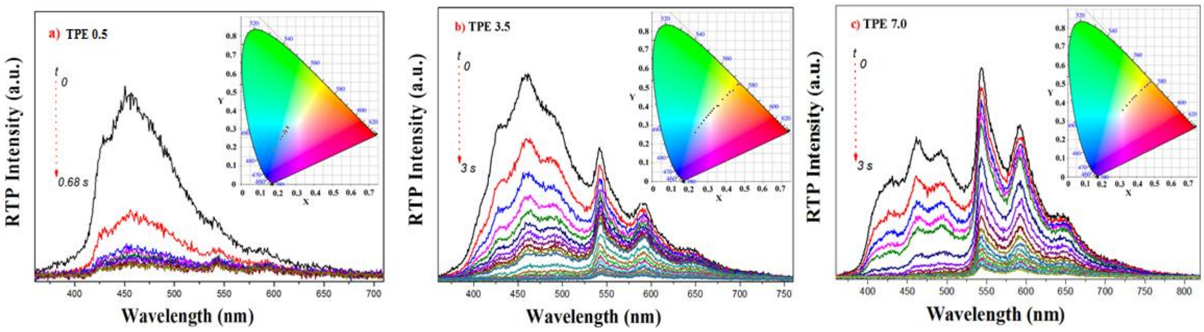

Figure S3. The delayed RTP spectra and the corresponding CIE 1931 chromaticity coordinates of photo-activated TPEx after 365 nm light excitation. A clear boundary at ca. 525 nm is observed, and the emissions before and after 525 nm are from mono-molecules and micro-crystals of PCN in SIS matrix, respectively.

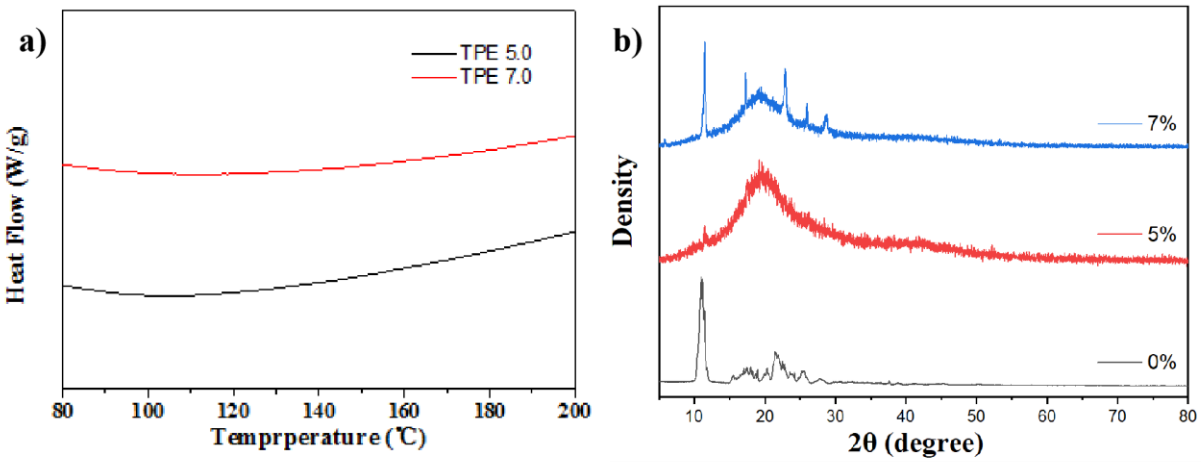

Figure S4. Differential scanning calorimetric (DSC) and X-ray diffraction (XRD) curves of TPEx (x=0, 5.0, 7.0).

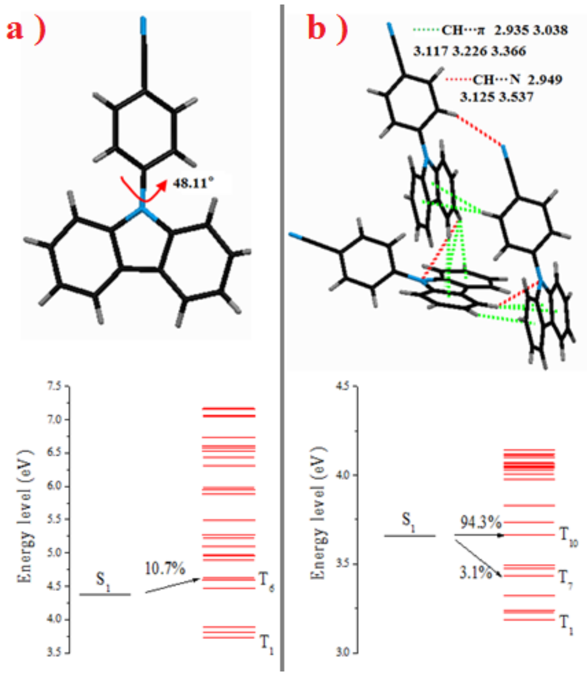

Figure S5. Possible ISC channels of PCN for a) a single molecule and b) three adjacent molecules.

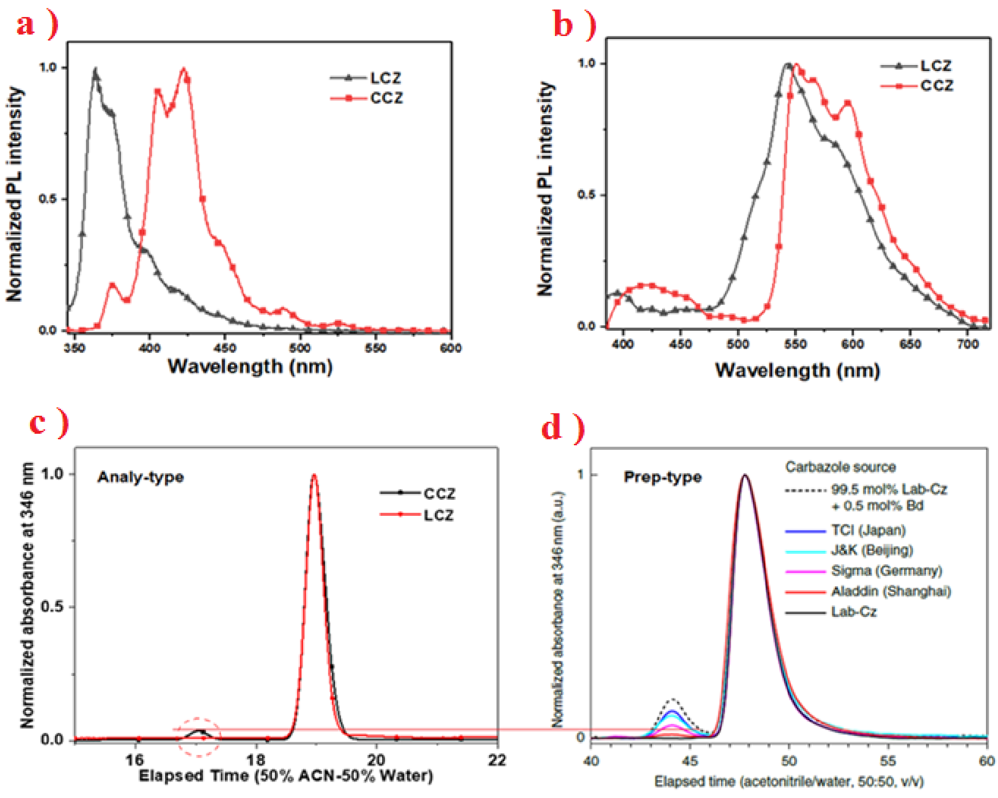

Figure S6. (a) The prompt spectra of LCZ and CCZ crystals with 340 nm excitation under common ambient conditions. (b) The delay spectra of LCZ and CCZ crystals with 360 nm excitation under common ambient conditions. RTP afterglow photographs of LCZ (c) HPLC spectra of CCZ and LCZ monitored at the onset absorption of 346 nm with 50/50 acetonitrile (ACN)-water ratio (v/v). (d) HPLC spectra of different carbazole source monitored at the onset absorption of 346 nm with 50/50 acetonitrile (ACN)-water ratio (v/v) as the eluent reported by Liu et al.[1] By comparing, the content of 1H-benzo[f]indole in the CCZ we used is about 0.2%.

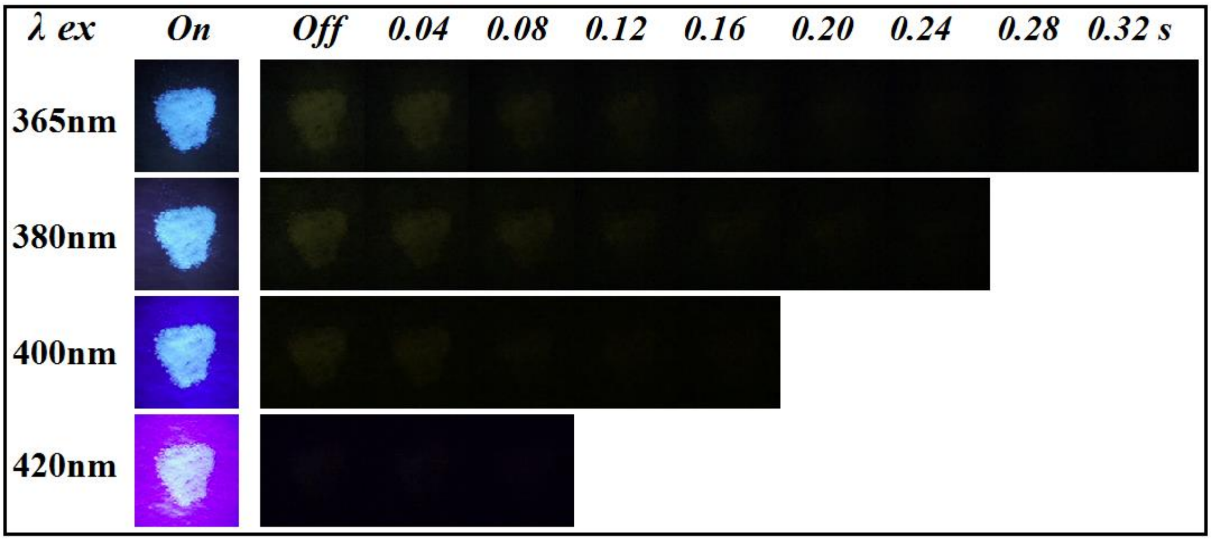

Figure S7. The PL photographs of L-PCN crystal before and after removing exaction light in the dark.

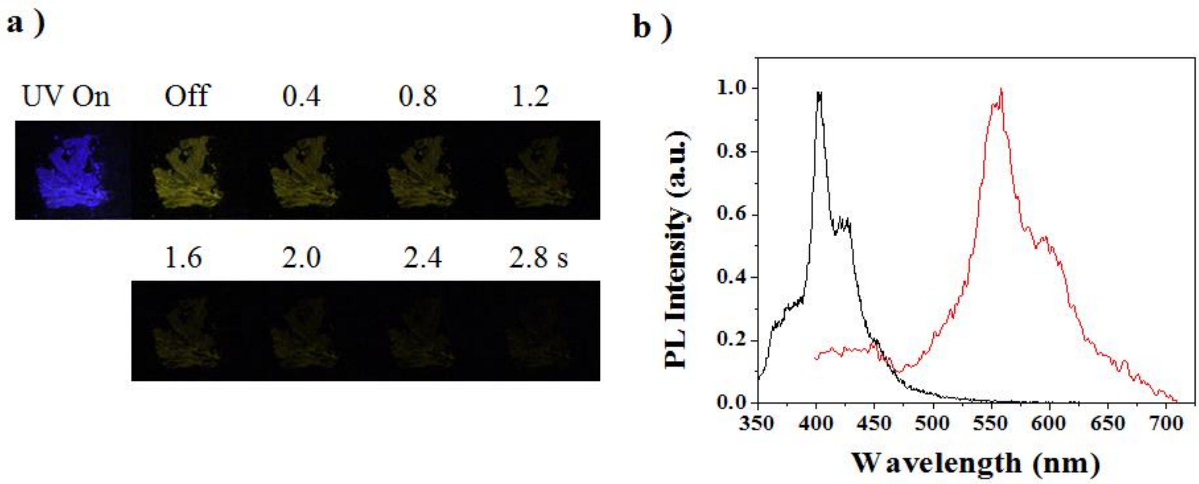

Figure S8. The PL photographs of PCN crystal and the prompt and delayed PL spectra of the pure PCN crystal be treated under 160 °C as the films.

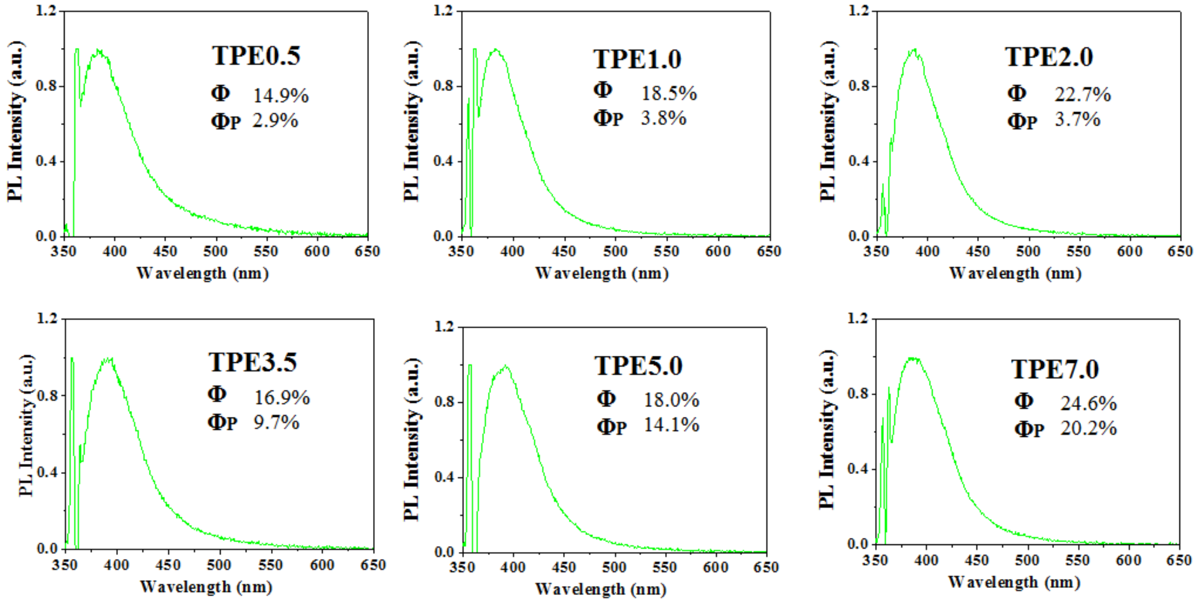

Figure S9. The RTP efficiencies of photo-activated TPEx measured at room temperature under 360 nm excitation.

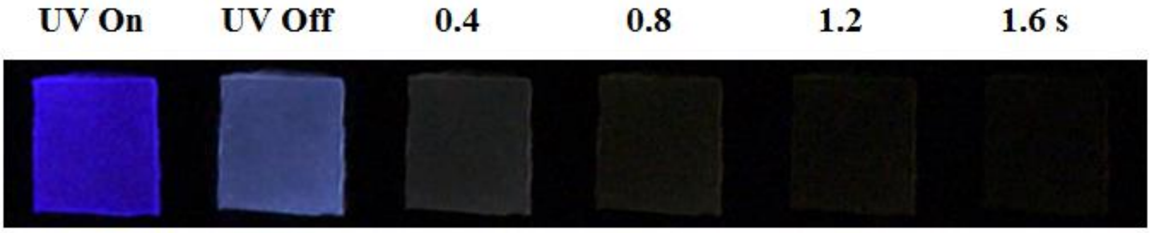

Figure S10. The PL photographs of TPE3.5 from direct solid thermoplasticized.

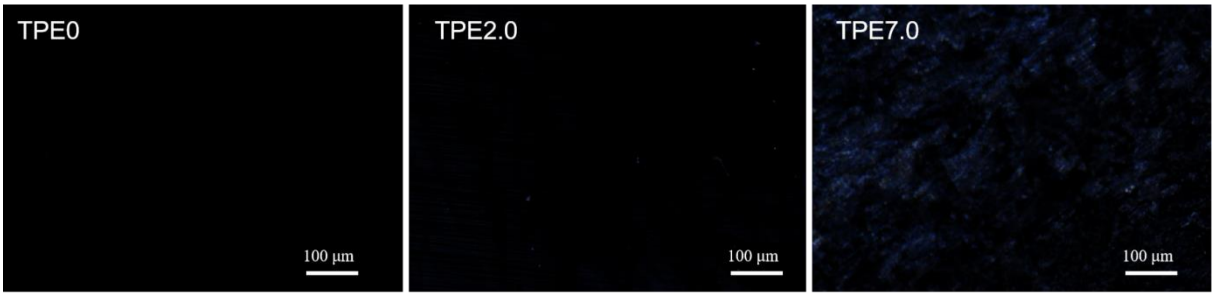

Figure S11. The polarizing microscope photographs of TPEx(x=0, 2.0, 7.0).

Table S1. The singlet and triplet excited state transition configurations of the PCN from single crystal revealed by TD-DFT calculations. The matched excited states that contain the same orbital transition components of S_1 and $|S_1 - T_n| < 0.3$ eV were highlighted in red.

	n	Energy	Orbitals	Transition

S_n	S_1	4.3802 eV	HOMO -> LUMO	0.688
T_n	T_1	3.7236 eV	HOMO-4 -> LUMO+2	0.028
			HOMO-3 -> LUMO	0.220
			HOMO -> LUMO	0.679
	T_2	3.8057 eV	HOMO-5 -> LUMO+5	0.037
			HOMO-2 -> LUMO+1	0.029
			HOMO-1 -> LUMO+1	0.669
			HOMO -> LUMO+1	0.113
			HOMO -> LUMO+3	0.069
	T_3	3.8935 eV	HOMO-3 -> LUMO+1	0.020
			HOMO-2 -> LUMO+3	0.034
			HOMO-2 -> LUMO+5	0.028
			HOMO-1 -> LUMO+1	0.103
			HOMO -> LUMO+1	0.767
	T_4	4.4656 eV	HOMO-2 -> LUMO+1	0.144
			HOMO-2 -> LUMO+4	0.052
			HOMO-1 -> LUMO+1	0.148
			HOMO -> LUMO+2	0.035
			HOMO -> LUMO+3	0.545
	T_5	4.5946 eV	HOMO-3 -> LUMO+2	0.188
			HOMO -> LUMO+2	0.688
			HOMO -> LUMO+3	0.026
	T_6	4.6337 eV	HOMO-5 -> LUMO	0.021
			HOMO-4 -> LUMO+2	0.157
			HOMO-3 -> LUMO	0.280
			HOMO-2 -> LUMO+3	0.089
			HOMO-1 -> LUMO+5	0.020
			HOMO -> LUMO	0.107
			HOMO -> LUMO+4	0.208
	T_7	4.9002 eV	HOMO-5 -> LUMO+3	0.032
			HOMO-2 -> LUMO+1	0.117
			HOMO-1 -> LUMO	0.583
			HOMO-1 -> LUMO+3	0.030
			HOMO-1 -> LUMO+4	0.136
			HOMO -> LUMO+5	0.049
	T_8	4.9616 eV	HOMO-5 -> LUMO+1	0.034
			HOMO-4 -> LUMO	0.059
			HOMO-4 -> LUMO+2	0.490
			HOMO-2 -> LUMO+3	0.071
			HOMO-1 -> LUMO+5	0.038
			HOMO -> LUMO	0.048
			HOMO -> LUMO+4	0.174
	T_9	4.9784 eV	HOMO-5 -> LUMO+1	0.154
			HOMO-5 -> LUMO+4	0.022
			HOMO-1 -> LUMO+3	0.690
			HOMO-1 -> LUMO+5	0.044
	T_{10}	5.0988 eV	HOMO-4 -> LUMO	0.860
			HOMO-4 -> LUMO+2	0.059
			HOMO -> LUMO+2	0.030
	T_{11}	5.2176 eV	HOMO-4 -> LUMO+2	0.055
			HOMO-3 -> LUMO	0.068
			HOMO-2 -> LUMO+1	0.296
			HOMO-2 -> LUMO+4	0.031
			HOMO-1 -> LUMO	0.157
			HOMO -> LUMO	0.022
			HOMO -> LUMO+3	0.103
			HOMO -> LUMO+5	0.166
	T_{12}	5.2712 eV	HOMO-6 -> LUMO	0.028

			HOMO-5 -> LUMO+1	0.052
			HOMO-4 -> LUMO+2	0.145
			HOMO-3 -> LUMO	0.245
			HOMO-2 -> LUMO+1	0.068
			HOMO-2 -> LUMO+3	0.021
			HOMO-1 -> LUMO	0.056
			HOMO-1 -> LUMO+3	0.055
			HOMO-1 -> LUMO+5	0.075
			HOMO -> LUMO	0.068
			HOMO -> LUMO+3	0.025
			HOMO -> LUMO+4	0.031
			HOMO -> LUMO+5	0.032
	T_{13}	5.4865 eV	HOMO-5 -> LUMO	0.026
			HOMO-5 -> LUMO+1	0.172
			HOMO-3 -> LUMO	0.026
			HOMO-2 -> LUMO+3	0.060
			HOMO-1 -> LUMO+3	0.102
			HOMO-1 -> LUMO+5	0.255
			HOMO -> LUMO+4	0.220
	T_{14}	5.8832 eV	HOMO-3 -> LUMO+2	0.150
			HOMO-1 -> LUMO+2	0.699
			HOMO -> LUMO+2	0.061
	T_{15}	5.9458 eV	HOMO-5 -> LUMO+2	0.024
			HOMO-3 -> LUMO+2	0.516
			HOMO-1 -> LUMO+2	0.195
			HOMO-1 -> LUMO+4	0.035
			HOMO -> LUMO+2	0.125
	T_{16}	5.9863 eV	HOMO-5 -> LUMO+3	0.096
			HOMO-2 -> LUMO	0.096
			HOMO-1 -> LUMO	0.103
			HOMO-1 -> LUMO+2	0.069
			HOMO-1 -> LUMO+4	0.539
	T_{17}	6.3132 eV	HOMO-2 -> LUMO	0.665
			HOMO-2 -> LUMO+1	0.031
			HOMO-2 -> LUMO+4	0.032
			HOMO-1 -> LUMO	0.029
			HOMO-1 -> LUMO+4	0.077
			HOMO -> LUMO+3	0.110
	T_{18}	6.4275 eV	HOMO-10 -> LUMO	0.225
			HOMO-9 -> LUMO	0.027
			HOMO-8 -> LUMO+6	0.030
			HOMO-7 -> LUMO+6	0.130
			HOMO-6 -> LUMO	0.101
			HOMO-3 -> LUMO	0.035
			HOMO-3 -> LUMO+4	0.024
			HOMO-3 -> LUMO+7	0.096
			HOMO-2 -> LUMO+3	0.036
			HOMO -> LUMO+4	0.056
	T_{19}	6.5266 eV	HOMO-3 -> LUMO+1	0.026
			HOMO-2 -> LUMO	0.065
			HOMO-2 -> LUMO+1	0.164
			HOMO-2 -> LUMO+4	0.022
			HOMO -> LUMO+3	0.032
			HOMO -> LUMO+5	0.526
	T_{20}	6.5742 eV	HOMO-10 -> LUMO	0.023
			HOMO-6 -> LUMO+1	0.040
			HOMO-3 -> LUMO+1	0.129
			HOMO-2 -> LUMO+1	0.023
			HOMO-2 -> LUMO+3	0.250

			HOMO-2 -> LUMO+5	0.020
			HOMO-1 -> LUMO+5	0.096
			HOMO -> LUMO+1	0.037
			HOMO -> LUMO+4	0.140
			HOMO -> LUMO+5	0.044
	T_{21}	6.6095 eV	HOMO-10 -> LUMO+6	0.023
			HOMO-8 -> LUMO	0.119
			HOMO-7 -> LUMO	0.545
			HOMO-7 -> LUMO+4	0.024
			HOMO-7 -> LUMO+7	0.080
			HOMO-6 -> LUMO	0.025
			HOMO-3 -> LUMO+6	0.053
	T_{22}	6.7366 eV	HOMO-6 -> LUMO+1	0.048
			HOMO-6 -> LUMO+4	0.038
			HOMO-5 -> LUMO	0.034
			HOMO-5 -> LUMO+1	0.027
			HOMO-3 -> LUMO+1	0.243
			HOMO-3 -> LUMO+4	0.026
			HOMO-2 -> LUMO+3	0.179
			HOMO-2 -> LUMO+5	0.104
			HOMO-1 -> LUMO+5	0.110
			HOMO -> LUMO+4	0.065
	T_{23}	7.0531 eV	HOMO-10 -> LUMO+6	0.023
			HOMO-3 -> LUMO+6	0.085
			HOMO-2 -> LUMO+2	0.720
			HOMO -> LUMO+6	0.046
	T_{24}	7.0601 eV	HOMO-10 -> LUMO+6	0.079
			HOMO-7 -> LUMO	0.071
			HOMO-6 -> LUMO+6	0.054
			HOMO-5 -> LUMO+6	0.025
			HOMO-3 -> LUMO+6	0.300
			HOMO-2 -> LUMO+2	0.209
			HOMO -> LUMO+6	0.163
	T_{25}	7.1580 eV	HOMO-5 -> LUMO	0.594
			HOMO-5 -> LUMO+1	0.088
			HOMO-3 -> LUMO	0.033
			HOMO-2 -> LUMO+3	0.031
			HOMO-1 -> LUMO+3	0.023
			HOMO-1 -> LUMO+5	0.023
	T_{26}	7.1673 eV	HOMO-6 -> LUMO+3	0.044
			HOMO-5 -> LUMO+3	0.095
			HOMO-3 -> LUMO+3	0.158
			HOMO-2 -> LUMO	0.077
			HOMO-2 -> LUMO+1	0.032
			HOMO-2 -> LUMO+4	0.330
			HOMO-1 -> LUMO+4	0.022
			HOMO -> LUMO+3	0.040
			HOMO -> LUMO+5	0.068

Table S2. The singlet and triplet excited state transition configurations of the PCN from single crystal revealed by TD-DFT calculations. The matched excited states that contain the same orbital transition components of S_1 and $|S_1 - T_n| < 0.3$ eV were highlighted in red[2].

	n	Energy	Orbitals	Transition

S_n	S_1	3.6573 eV	HOMO -> LUMO+1	0.990
T_n	T_1	3.1875 eV	HOMO-13 -> LUMO+7	0.021
			HOMO-10 -> LUMO+1	0.112
			HOMO-1 -> LUMO+1	0.788
	T_2	3.2256 eV	HOMO-15 -> LUMO+4	0.025
			HOMO-11 -> LUMO	0.138
			HOMO-2 -> LUMO	0.761
	T_3	3.2404 eV	HOMO-12 -> LUMO+8	0.040
			HOMO-9 -> LUMO+2	0.123
			HOMO -> LUMO+2	0.721
			HOMO -> LUMO+5	0.036
	T_4	3.3204 eV	HOMO-14 -> LUMO+16	0.026
			HOMO-7 -> LUMO+6	0.027
			HOMO-7 -> LUMO+13	0.022
			HOMO-4 -> LUMO+4	0.274
			HOMO-4 -> LUMO+6	0.377
			HOMO-4 -> LUMO+7	0.023
			HOMO-1 -> LUMO+10	0.111
	T_5	3.3239 eV	HOMO-17 -> LUMO+15	0.031
			HOMO-8 -> LUMO+3	0.048
			HOMO-8 -> LUMO+12	0.025
			HOMO-5 -> LUMO+3	0.661
			HOMO-2 -> LUMO+3	0.038
			HOMO-2 -> LUMO+9	0.119
	T_6	3.3249 eV	HOMO-6 -> LUMO+5	0.044
			HOMO-6 -> LUMO+14	0.020
			HOMO-3 -> LUMO+5	0.597
			HOMO-3 -> LUMO+7	0.048
			HOMO -> LUMO+5	0.043
			HOMO -> LUMO+11	0.112
	T_7	3.4314 eV	HOMO-6 -> LUMO+11	0.030
			HOMO-3 -> LUMO+5	0.039
			HOMO -> LUMO+1	0.031
			HOMO -> LUMO+2	0.030
			HOMO -> LUMO+5	0.723
			HOMO -> LUMO+7	0.060
	T_8	3.4742 eV	HOMO-8 -> LUMO+9	0.033
			HOMO-5 -> LUMO+3	0.033
			HOMO-2 -> LUMO+3	0.843
	T_9	3.4952 eV	HOMO-7 -> LUMO+10	0.033
			HOMO-1 -> LUMO+4	0.365
			HOMO-1 -> LUMO+6	0.465
			HOMO-1 -> LUMO+7	0.023
	T_{10}	3.6638 eV	HOMO -> LUMO+1	0.943
			HOMO -> LUMO+5	0.040
	T_{11}	3.7330 eV	HOMO-1 -> LUMO	0.992
	T_{12}	3.8293 eV	HOMO -> LUMO	0.997
	T_{13}	3.9764 eV	HOMO-10 -> LUMO+7	0.025

			HOMO-4 -> LUMO+1	0.148
			HOMO-1 -> LUMO+2	0.030
			HOMO-1 -> LUMO+5	0.103
			HOMO-1 -> LUMO+7	0.583
	T_{14}	3.9796 eV	HOMO-10 -> LUMO+1	0.030
			HOMO-4 -> LUMO+1	0.664
			HOMO-1 -> LUMO+5	0.026
			HOMO-1 -> LUMO+7	0.149
	T_{15}	4.0090 eV	HOMO-11 -> LUMO	0.021
			HOMO-11 -> LUMO+4	0.028
			HOMO-2 -> LUMO+4	0.588
			HOMO-2 -> LUMO+6	0.236
	T_{16}	4.0322 eV	HOMO-16 -> LUMO+7	0.020
			HOMO-13 -> LUMO+7	0.077
			HOMO-10 -> LUMO+1	0.223
			HOMO-4 -> LUMO+1	0.064
			HOMO-3 -> LUMO+1	0.273
			HOMO-3 -> LUMO+2	0.031
			HOMO-1 -> LUMO+1	0.067
			HOMO-1 -> LUMO+13	0.022
			HOMO -> LUMO+8	0.030
			HOMO -> LUMO+11	0.027
	T_{17}	4.0403 eV	HOMO-13 -> LUMO+7	0.046
			HOMO-10 -> LUMO+1	0.138
			HOMO-4 -> LUMO+1	0.026
			HOMO-3 -> LUMO+1	0.259
			HOMO-3 -> LUMO+2	0.044
			HOMO-3 -> LUMO+5	0.034
			HOMO-1 -> LUMO+1	0.043
			HOMO -> LUMO+8	0.177
			HOMO -> LUMO+11	0.070
	T_{18}	4.0451 eV	HOMO-15 -> LUMO+4	0.095
			HOMO-15 -> LUMO+6	0.052
			HOMO-11 -> LUMO	0.294
			HOMO-5 -> LUMO	0.219
			HOMO-2 -> LUMO	0.108
			HOMO-2 -> LUMO+4	0.047
			HOMO-2 -> LUMO+12	0.043
	T_{19}	4.0527 eV	HOMO-12 -> LUMO+8	0.061
			HOMO-9 -> LUMO+2	0.124
			HOMO-9 -> LUMO+8	0.035
			HOMO-3 -> LUMO+1	0.123
			HOMO-3 -> LUMO+2	0.055
			HOMO -> LUMO+2	0.054
			HOMO -> LUMO+8	0.431
	T_{20}	4.0658 eV	HOMO-12 -> LUMO+8	0.146
			HOMO-9 -> LUMO+2	0.225
			HOMO-6 -> LUMO+11	0.020

			HOMO-3 -> LUMO+1	0.141
			HOMO -> LUMO+2	0.095
			HOMO -> LUMO+8	0.200
			HOMO -> LUMO+14	0.037
	T_{21}	4.0738 eV	HOMO-15 -> LUMO+4	0.031
			HOMO-11 -> LUMO	0.089
			HOMO-5 -> LUMO	0.504
			HOMO-5 -> LUMO+3	0.060
			HOMO-4 -> LUMO	0.158
			HOMO-2 -> LUMO	0.024
			HOMO-2 -> LUMO+9	0.040
	T_{22}	4.0998 eV	HOMO-11 -> LUMO	0.024
			HOMO-5 -> LUMO	0.085
			HOMO-4 -> LUMO	0.834
	T_{23}	4.1142 eV	HOMO-6 -> LUMO+5	0.035
			HOMO-3 -> LUMO+1	0.180
			HOMO-3 -> LUMO+2	0.161
			HOMO-3 -> LUMO+5	0.171
			HOMO -> LUMO+8	0.063
			HOMO -> LUMO+11	0.268
	T_{24}	4.1199 eV	HOMO-2 -> LUMO+1	0.979
	T_{25}	4.1429 eV	HOMO-8 -> LUMO+3	0.108
			HOMO-8 -> LUMO+12	0.026
			HOMO-5 -> LUMO	0.108
			HOMO-5 -> LUMO+3	0.137
			HOMO-2 -> LUMO+9	0.450
			HOMO-1 -> LUMO+3	0.033
			HOMO-1 -> LUMO+10	0.027
	T_{26}	4.1439 eV	HOMO-7 -> LUMO+4	0.034
			HOMO-7 -> LUMO+6	0.047
			HOMO-7 -> LUMO+13	0.021
			HOMO-4 -> LUMO+4	0.064
			HOMO-4 -> LUMO+6	0.071
			HOMO-3 -> LUMO+2	0.054
			HOMO-2 -> LUMO+9	0.034
			HOMO-1 -> LUMO+2	0.068
			HOMO-1 -> LUMO+3	0.045
			HOMO-1 -> LUMO+9	0.026
			HOMO-1 -> LUMO+10	0.368

NMR

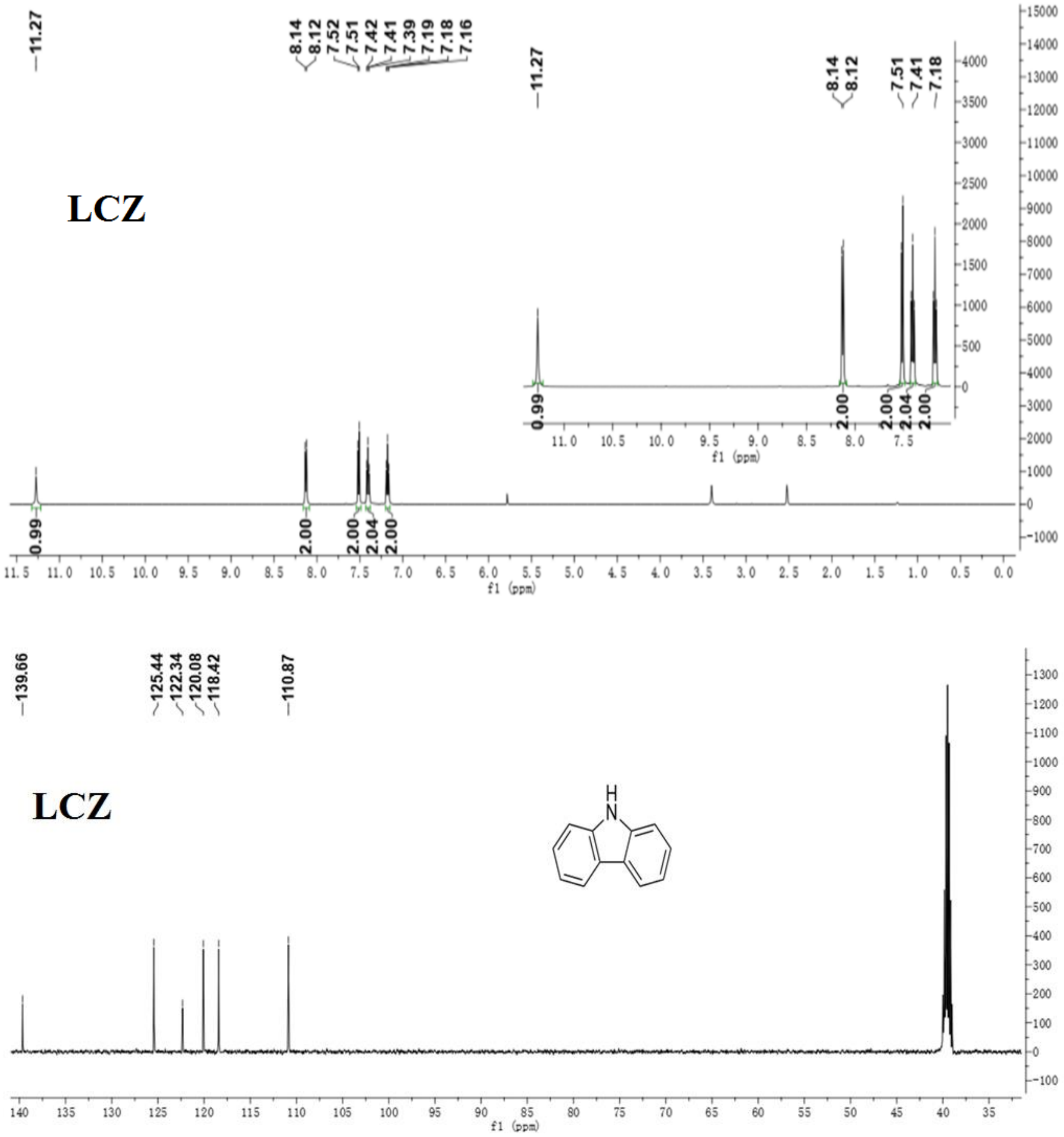

Figure S8. ^{1}H and ^{13}C NMR spectrum of LCZ in DMSO-d_6.

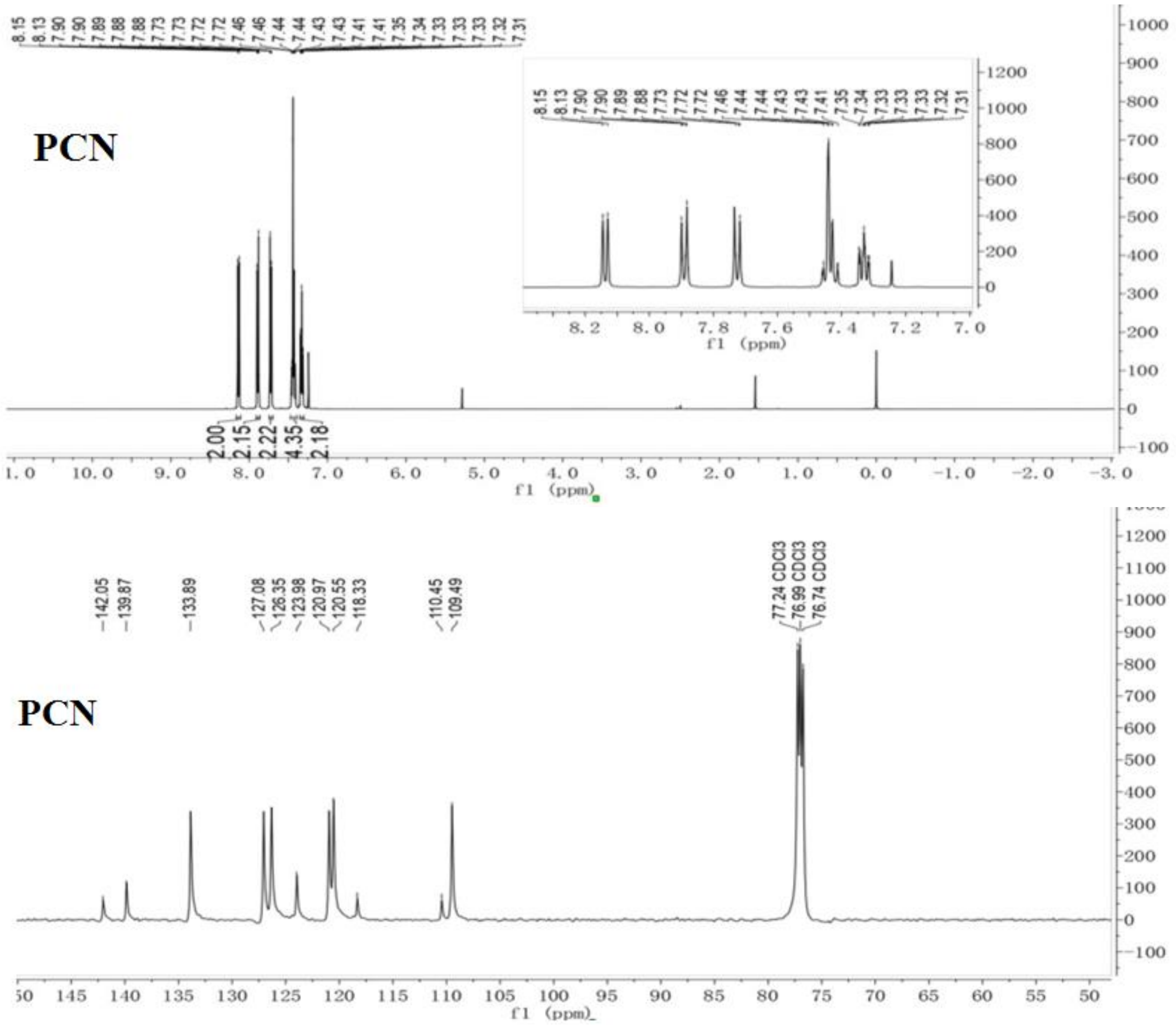

Figure S9. ^{1}H and ^{13}C NMR spectrum of PCN in $CDCl_3$.

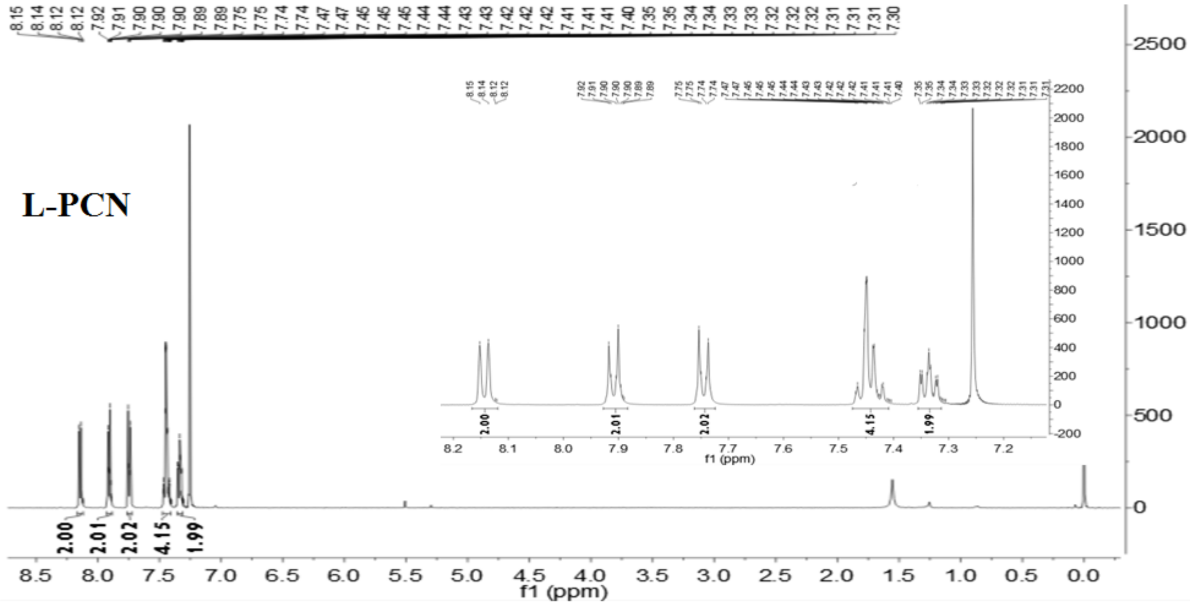

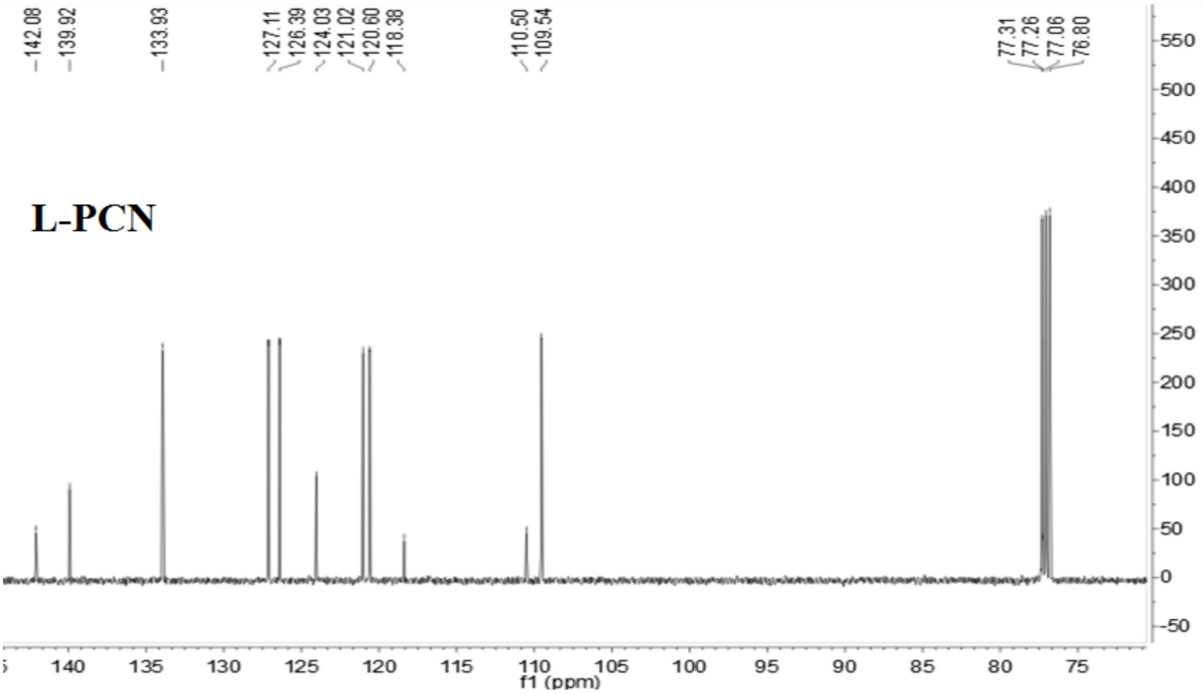

Figure S10. ^{1}H and ^{13}C NMR spectrum of L-PCN in $CDCl_3$.

References

[1] C. J. Chen, Z. G. Chi, K. C. Chong, A. S. Batsanov, Z. Yang, Z. Mao, Z. Y. Yang, B, Liu, *Nat. Mater.* **2021**, *20*, 175.

[2] Y. Wang, Z. Zhang, L. Liu, S. Yuan, J. Ma, D. Liu, S. Xue, Q. Sun, W. Yang, *J. Mater. Chem. C.* **2019**, *7*, 9671.
